# The AP-1 transcription factor homolog *Pf*-AP-1 activates transcription of multiple biomineral proteins and potentially participates in *Pinctada fucata* biomineralization

**DOI:** 10.1038/srep14408

**Published:** 2015-09-25

**Authors:** Xiangnan Zheng, Minzhang Cheng, Liang Xiang, Jian Liang, Liping Xie, Rongqing Zhang

**Affiliations:** 1 Institute of Marine Biotechnology, Collaborative Innovation Center of Deep Sea Biology, School of Life Sciences, Tsinghua University, Beijing 100084, China; 2 Protein Science Laboratory of the Ministry of Education, Tsinghua University, Beijing 100084, China

## Abstract

Activator protein-1 (AP-1) is an important bZIP transcription factor that regulates a series of physiological processes by specifically activating transcription of several genes, and one of its well-chartered functions in mammals is participating in bone mineralization. We isolated and cloned the complete cDNA of a *Jun/AP-1* homolog from *Pinctada fucata* and called it *Pf*-AP-1. *Pf*-AP-1 had a highly conserved bZIP region and phosphorylation sites compared with those from mammals. A tissue distribution analysis showed that *Pf*-AP-1 was ubiquitously expressed in *P. fucata* and the mRNA level of *Pf*-AP-1 is extremely high in mantle. *Pf*-AP-1 expression was positively associated with multiple biomineral proteins in the mantle. The luciferase reporter assay in a mammalian cell line showed that *Pf*-AP-1 significantly up-regulates the transcriptional activity of the promoters of KRMP, Pearlin, and Prisilkin39. Inhibiting the activity of Pf-AP-1 depressed the expression of multiple matrix proteins. *Pf*-AP-1 showed a unique expression pattern during shell regeneration and pearl sac development, which was similar to the pattern observed for biomineral proteins. These results suggest that the *Pf*-AP-1 AP-1 homolog is an important transcription factor that regulates transcription of several biomineral proteins simultaneously and plays a role in *P. fucata* biomineralization, particularly during pearl and shell formation.

Activator protein-1 (AP-1) is a ubiquitous and important transcription factor that participates in many physiological processes. AP-1 is a homodimeric or heterodimeric protein consisting of Jun family members (Jun, JunB, and JunD) and Fos family members (c-Fos, FosB, Fra1, and Fra2), and all of these members have a conserved bZIP domain^1^,^2^,^3^. AP-1 interacts with other bZIP proteins or cofactors and regulates cell differentiation, cell cycle progression, apoptosis, tumorigenesis, and bone mineralization^4^,^5^. The regulatory ability of AP-1 is cell or tissue-specific.

Jun, which was originally described as c-Jun or P39^3^,^6^, is the central component of all AP-1 complexes. As the first identified AP-1 component with similar structural and functional properties to those of AP-1, *Jun* was once regarded as the AP-1 gene^7^,^8^. Jun is also called Jun/AP-1 or AP-1, particularly in protein and antibody products^9^. There are many complex post-transcriptional modifications of Jun, which regulates Jun functions in cellular processes and diseases^10^. It is antagonistic with JunB during fibroblast, keratinocyte, and granulocyte differentiation. Jun promotes cell cycle progression and drives apoptosis in fibroblasts and neurons but has a negative function during hepatocyte and keratinocyte apoptosis^5^. Jun also plays an important role in bone cell differentiation, as Jun proteins rescue osteoclast differentiation in c-Fos-deficient precursors. Several studies have demonstrated that Jun is a key transcription factor during bone formation and reconstruction. Jun and its phosphorylated form are essential for efficient osteoclastogenesis^11^. Jun is recruited and activated by the receptor activator of nuclear factor kappa-B ligand (RANKL) in osteoclast precursors^12^,^13^. In addition, the signaling pathways mediated by RANK/AP-1 are critical for osteoclastogenesis^14^. Inhibiting Jun N-terminal kinase (JNK) in synovial fibroblasts suppresses the increases in phospho-Jun and expression of the interstitial collagenase gene^15^. Phospho-Jun inhibits transforming growth factor-β-induced *COL1A2* gene expression^16^. Jun also responds to mechanical compression loading in cartilage^17^. Mitogen-activated protein kinase (MAPK) and AP-1 signaling improve *in vitro* cartilage formation^18^, and blocking AP-1 binding prevents increased expression of type II collagen and synthesis and accumulation of the matrix. Thus, Jun/AP-1 plays an important role in mammalian bone formation.

Most studies on AP-1 have focused on mammals and model organisms. In mollusks, AP-1 has only been identified from abalone, *Haliotis discus discus*^19^, Hong Kong oyster, *Crassostrea hongkongensis*^20^, and the Manila clam, *Venerupis philippinarum*^21^. Furthermore, most studies on molluscan AP-1 have focused on immune function. However, the functions of molluscan biominerals, such as bivalve pearls and shells, have attracted attention because of their excellent mechanical properties^22^,^23^, precise structure^24^, and use in medical devices^25^,^26^,^27^. The structures and formation of pearls and shells are accurately regulated by biomineral proteins. The functions of more than 20 biomineral proteins have been studied, but little is known about the regulatory mechanism for their expression. *Pinctada fucata*, an economically important bivalve species and one of the main pearl producers in China, is a popular molluscan species for biomineralization research. Few transcription factors in *P. fucata* had been reported, and PfMSX is the only one which had been well-studied with transcription activity checked^28^. Human AP-1 has been reported with regulatory function in *P. fucata* matrix protein transcription *in vitro*, but the existence and sequence of AP-1 homologs in *P. fucata* remains unknown, and more importantly, the function of *P. fucata* AP-1 protein *in vivo* had little been illustrated yet^29^. Thus, we cloned and characterized the complete cDNA sequence of AP-1 from *P. fucata* and performed a series of analyses to investigate whether AP-1 could influence pearl and shell formation by regulating biomineral protein transcription. Protein sequence alignment and phylogenetic analyses revealed that the AP-1 protein was conserved from invertebrates to mammals, and a tissue distribution analysis revealed its ubiquitous expression. We used the HEK293T cell line as a detection system. The transactivation ability of *Pf*-AP-1 for the KRMP, Pearlin, and Prisilkin39 promoter was analyzed using a luciferase reporter assay. The AP-1 and biomineral protein gene expression patterns during shell regeneration and pearl sac development were determined by quantitative reverse transcription-polymerase chain reaction (qRT-PCR) analysis.

## Results

### Cloning and characterization of the *Pf-AP-1* complete coding sequence (CDS)

**Figure 1:**
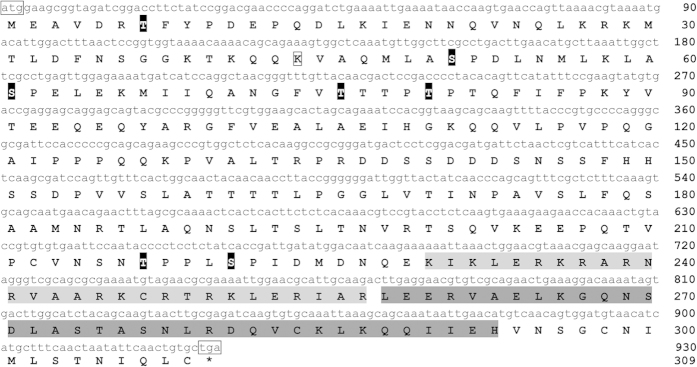
cDNA sequence and deduced amino acid sequence of *Pinctada fucata* activator protein-1 (AP-1). The start and stop codons are boxed; the asterisk at the end of the amino acid sequence indicates the stop codon. The basic motif is shaded light gray, and the leucine-zipper is shaded dark gray. The potential phosphorylation sites are shaded black, and the potential acetylation site is boxed.

We isolated the cDNA of the c-Jun homolog by PCR and named it *Pf*-AP-1. The cDNA was 1,361 bp long, with a 51-bp 5′-untranslated region (UTR) and a 380-bp 3′-UTR with a poly-A tail (GenBank accession no: KP347629). The open reading frame (ORF) was 930 bp and encoded a 309-amino acid (aa) bZIP family protein (Fig. 1). As a hallmark, a base domain and a leucine-zipper were identified in the C-terminal region (aa 230–293), which is highly conserved among mammals, and shaded in Fig. 1. Many of the modified *Pf*-AP-1 residues were highly conserved compared with those of human AP-1, particularly residues phosphorylated by p21 protein-activated kinase 2 (PAK2; aa 7, 76, and 80), MAPK8 (aa 51 and 61), polo-like kinase 3 (PLK3; aa 51 and 61), dual-specificity tyrosine-(Y)-phosphorylation-regulated kinase 2 (DYRK2; aa 221), and glycogen synthase kinase 3-beta (GSK3-β; aa 217 and 221), whereas aa 44 may be a potential acetylation site. These residues are shaded or boxed in Fig. 1. Thus, *Pf*-AP-1 was very similar to homologs of other species.

**Figure 2:**
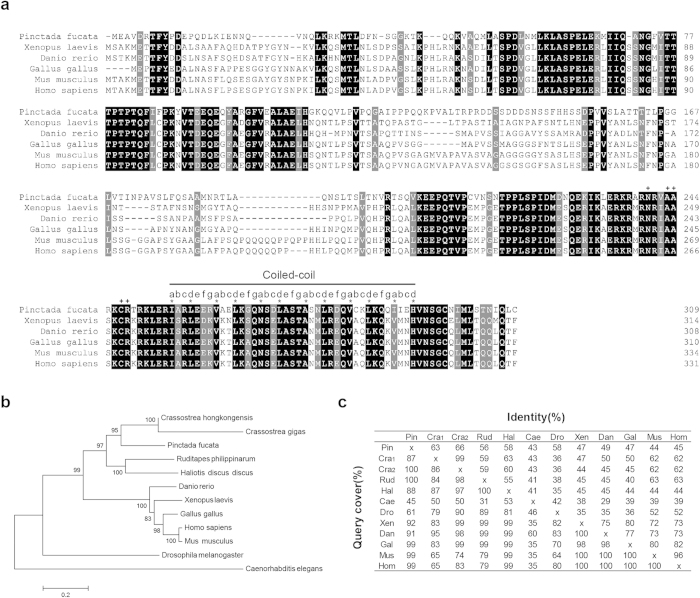
(**a**) Protein sequence alignment. The results were processed at http://www.bio-soft.net/sms/index.html. Their GenBank accession numbers are: *Pinctada fucata* (KP347629), *Xenopus laevis* (AAH74377), *Danio rerio* (NP 956281), *Gallus gallus* (AAA48927), *Mus musculus* (NP 034721), and *Homo sapiens* (NP 002219). The black bar indicates the coiled-coil in the bZIP domain. The a-g heptad repeat is displayed above the sequences. Residues that form the hydrophobic interface are marked with an asterisk. Residues that contact the DNA bases are marked with a plus sign (+). (**b**) Phylogenetic analysis of *P. fucata* activator protein-1 (*Pf*-AP-1) with the AP-1 proteins from other species. The phylogenetic tree was reconstructed from alignment using the neighbor joining (NJ) method in MEGA 5.22. The amino acid sequences are the same as those in (**a**). The other amino acid sequences are: *Crassostrea hongkongensis* (KC890768), *Crassostrea gigas* (EKC41210), *Ruditapes philippinarum* (HQ918289), *Haliotis discus discus* (ADQ43242), *Drosophila melanogaster* (CAA73154), and *Caenorhabditis elegans* (CAB76416). (**c**) Alignment of *Pf*-AP-1 with the AP-1 proteins from other species. The results were calculated using NCBI blastp. The upper right establishes the identity, and the lower left establishes the query cover as percentages. The amino acid sequences are the same as those in (**b**). Pin, *Pinctada* fucata; Cra1, *Crassostrea hongkongensis*; Cra2, *Crassostrea gigas*; Rud, *Ruditapes philippinarum*; Hal, *Haliotis discus discus*; Cae, *Caenorhabditis elegan*; Dro, *Drosophila melanogaster*; Xen, *Xenopus laevis*; Dan, *Danio rerio*; Gal, *Gallus gallus*; Mus, *Mus musculus*; Hom, *Homo sapiens*.

We aligned the protein sequences of *Pf*-AP-1 and those from model animals (Fig. 2a). The results showed that the C-terminal was highly conserved among species, and its secondary structure was similar to that of human AP-1. Seven residues within aa 255–292 formed a coil marked by an a-g heptad repeat. The residues at the a and d sites were responsible for the hydrophobic interface, which was conserved throughout all species. The residues in the base region contacting the DNA bases were also conserved and are marked with a plus (+) in Fig. 2a.

The close relationships among invertebrate and vertebrate AP-1 proteins were further confirmed by phylogenetic analysis. We constructed a phylogenetic tree using twelve representative species, including *P. fucata* and four other bivalves (Fig. 2b). The tree showed that the AP-1 protein evolved with the species. The AP-1 proteins from Nematoda and Arthropoda stood alone as two separate branches, and those from Mollusca were in a neighboring vertebrate branch. However, the vertebrate AP-1 was constructed from the main group. In this group, Amphibia, Pisces, Aves, and Mammalia had their own branches, and the distance to *Homo sapiens* decreased as species evolved.

We calculated the identities of the AP-1 proteins from invertebrates and vertebrates (Fig. 2c). As a result, the AP-1 proteins from *P. fucata* and *C. hongkongensis* were highly conserved (query included 100%; identity, 66%). *Pf*-AP-1 was more closely related to the AP-1 from mammals compared with those from other bivalves and reflected higher query coverage and a higher percentage identity. This result was confirmed by phylogenetic distance (Fig. 2b). These results suggest conservation of the *Pf*-AP-1 protein function compared with those from mammals.

### Tissue distribution of *Pf-AP-1* expression and the relevance of *Pf-AP-1* and biomineral proteins in oysters

**Figure 3:**
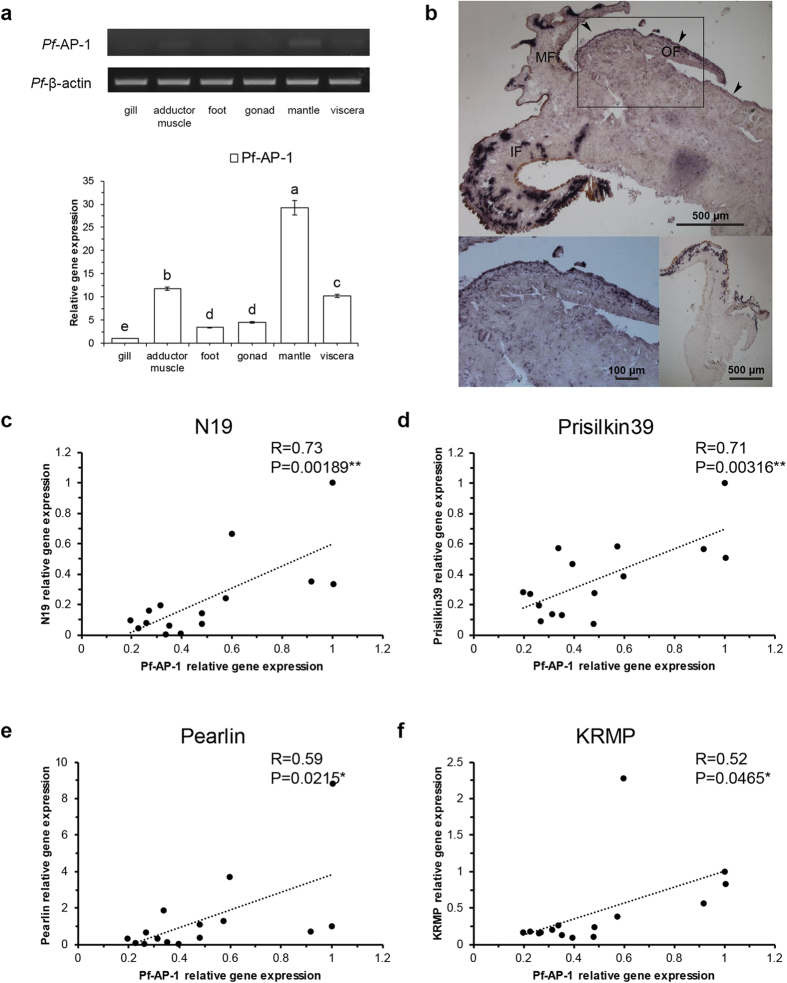
(**a**) Tissue distribution of *Pinctada fucata* activator protein-1 (*Pf*-AP-1) gene expression in *P. fucata*. Relative *Pf*-AP-1 mRNA expression in tissues was assessed by semi-quantitative and quantitative reverse transcription-polymerase chain reaction (qRT-PCR) analyses. Upper panel shows the semi-quantitative RT-PCR results, and the lower panel shows the qRT-PCR results. The results were analyzed by Duncan’s new multiple range method. Different superscript means significantly different (p < 0.05). (**b**)Detection of *Pf*-AP-1 mRNA in the mantle of *P. fucata* by *in-situ* hybridization. Hybridization signals (dark purple) in the inner epithelial cells of the outer fold and the outer epithelial cells of mantle are indicated by arrowheads in upper. OF, outer fold; MF, middle fold; IF, inner fold. The lower left showed the enlargement of the box in upper. The lower right showed the control section stained with the sense probe. (**c-f**)Relevance of *Pf-AP-1* and *N19*, *Prisilkin39*, *Pearlin*, and *KRMP* mRNA expression in normal oyster (n = 15), respectively, as analyzed by t-test.

**Figure 4:**
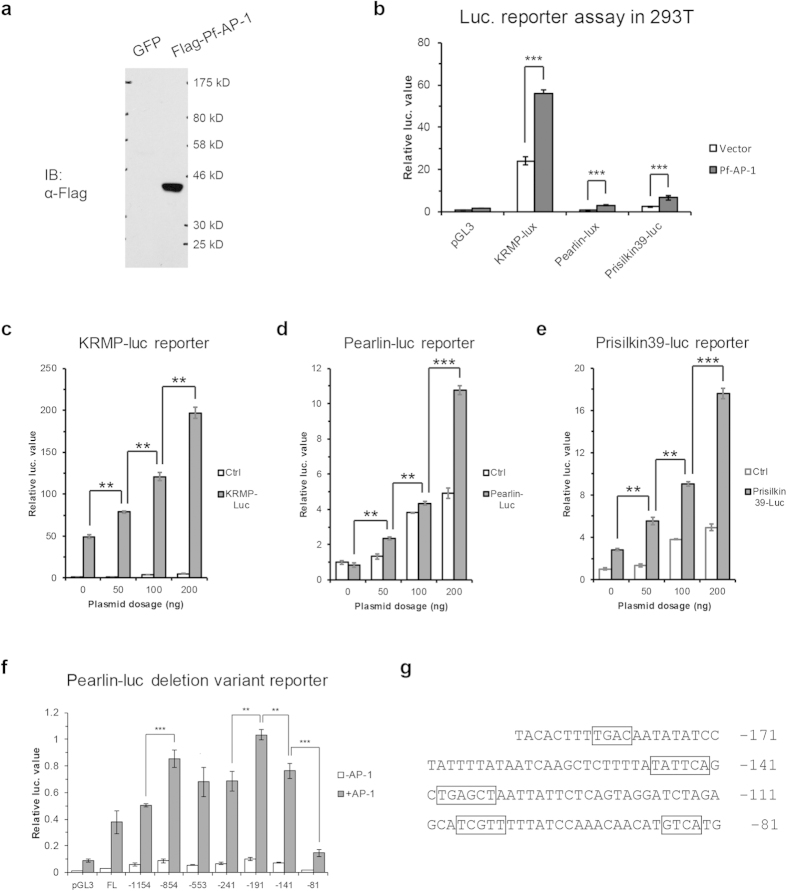
(**a**) Western blot assay of *Pinctada fucata* activator protein-1 (*Pf*-AP-1). The FLAG tagged recombined proteins were expressed in HEK293T cells. The green fluorescent protein (GFP) was used as a blank control. (**b**) Luciferase reporter assay to assess the ability of *Pf*-AP-1 to activate transcription of the KRMP, Pearlin, and Prisilkin39 promoter. The pGL3basic vector was used as a blank control. The results were analyzed by t-test. Three asterisks means p < 0.0001. (**c–e**) Luciferase reporter assay to assess the ability of different concentrations of *Pf*-AP-1 to activate the transcription of KRMP, Pearlin, and Prisilkin39. Each matrix proteins promoter was co-transfected with 0, 50, 100, or 200 ng of the FLAG-AP-1-pcDNA3.1 plasmid. The results were analyzed by t-test. Two asterisks means p < 0.01, and three asterisks means p < 0.0001. (**f**) Luciferase reporter assay of Pearlin promoter deletion variant in the present or absent of *Pf*-AP-1. The Pearlin promoter had a series of 5′ deletion. And the numbers under abscissa showed the start site of each promoter segment inserted in pGL3 basic. The results were analyzed by t-test. Two asterisks means p < 0.01, and three asterisks means p < 0.0001. (**g**) Putative AP-1 binding sites in Pearlin promoter predicted by Transcription Element Search System (TESS). Putative binding sites were boxed.

The *Pf*-AP-1 spatio-temporal expression pattern was analyzed by semi-quantitative RT-PCR and qRT-PCR. The Duncan’s new multiple range method was used to measure the significance of the expression in different tissues. An *in vivo* investigation revealed that *Pf*-AP-1 was ubiquitously expressed in all adult oyster tissues examined, with high levels detected in the adductor muscle and viscera. The highest *Pf*-AP-1 level was found in the mantle and it was significant, whereas relatively low levels were detected in the gill, foot, and gonad (Fig. 3a). The highest *Pf*-AP-1 expression in biomineral tissues suggests that *Pf*-AP-1 plays a role in regulating biomineralization. Furthermore, *in-situ* hybridization was performed to analyze the location of *Pf*-AP-1 in mantle. The result showed that hybridization signal arose in the whole tissue, which suggested that *Pf*-AP-1 is ubiquitously expressed in mantle (Fig. 4b). Strong hybridization signal (darker purple) was detected in inner epithelial cells and outer epithelial cells in mantle, where expressed most of the matrix proteins. Those results suggest that *Pf*-AP-1 had the same location with matrix proteins in expression, and *Pf*-AP-1 might have important function in regulating matrix proteins.

We detected gene expression of matrix proteins with that of *Pf*-AP-1 in 15 adult individuals. The qRT-PCR results and statistical analysis showed that expression of the matrix proteins Pearlin and KRMP was significantly correlated with *Pf*-AP-1 gene expression (P < 0.05), and N19 and Prisilkin39 expression was highly significantly correlated with that of *Pf*-AP-1 (P < 0.01) (Fig. 3c–f). These results suggest that *Pf*-AP-1 may regulate transcription of these matrix proteins.

### *Pf*-AP-1 transactivation ability

We subcloned the *Pf*-AP-1 coding DNA sequence into pcDNA3.1 containing a FLAG tag to construct the FLAG-AP-1-pcDNA3.1 expression vector to confirm correct expression of *Pf*-AP-1 in a eukaryotic system. We transfected the FLAG-AP-1-pcDNA3.1 expression vector into HEK293T cells to detect expression in eukaryotic cells, together with the green fluorescent protein (GFP) (Fig. 4a). Interestingly, immunoblotting (western blot) showed that the actual molecular weight of the *Pf*-AP-1 protein was approximately 42 kDa, which was much larger than the calculated molecular weight of 34.4 kDa. This result is similar to that of human AP-1, in which the actual molecular weight was larger than the theoretical weight. As mentioned before, the band shift may have resulted from post-transcriptional modifications, particularly multiple phosphorylations.

We used a luciferase reporter assay to measure transactivation ability of *Pf*-AP-1. We found that *Pf*-AP-1 transactivated the KRMP, Pearlin, and Prisilkin39 promoter in a dose-dependent manner (Fig. 4b–e).

To confirm the transactivation ability of *Pf*-AP-1, we make the deletion variant of Pearlin promoter. We cloned Pearlin promoter with a series of 5′ deletion promoter luciferase constructs and tested their transcriptional activity in HEK293T cells in the present or absent of *Pf*-AP-1 vector. Deletions of the region from −1154 to −854 bp and −241 to −191 bp resulted significantly promoter activity increase, which suggested these regions function as silencers in controlling Pearlin gene transactivation. Deletions of the region from −191 to −141 bp and −141 to −81 bp resulted significantly promoter activity decrease; the latter even made the promoter activity dropped to nearly basal level (pGL3) (Fig. 4f). These results confirmed the *Pf*-AP-1 transcriptional activity to Pearlin promoter, and suggested there were AP-1 functional sites in region −191 to −81 bp, while the region −141 to −81 bp might contained more important binding sites.

We analyzed the Pearlin promoter by Transcription Element Search System (TESS).Multiple putative AP-1 binding sites were found in the region from −191 to −81 bp, which took the most responsibility to the transcriptional activity (Fig. 4g). The promoter analysis indicated that *Pf*-AP-1 might bind to the promoter of Pearlin at the −191 to −81 bp region and initiate its transcription.

The promoters of KRMP and Prisilkin39 were also truncated for transcriptional activity test, and then analyzed by TESS, which showed similar results with that of Pearlin (data not shown).

### *In vivo* AP-1 inhibition experiment

**Figure 5:**
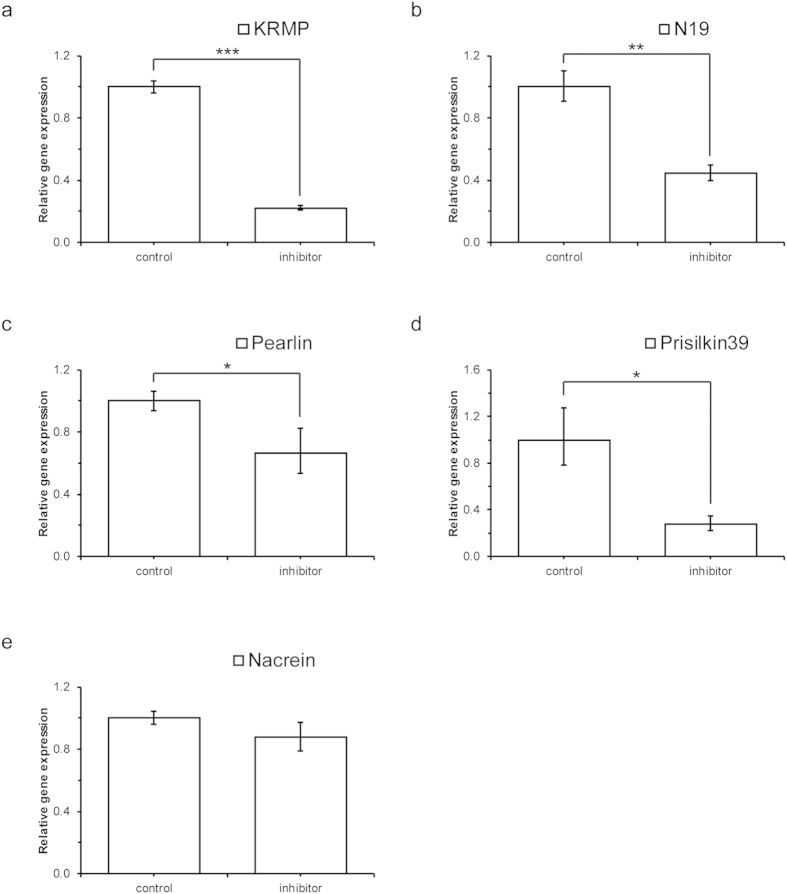
Relative mRNA expression of matrix proteins under AP-1 inhibitior. Quantitative reverse transcription-polymerase chain reaction (qRT-PCR) analysis was performed to evaluate the expression of each gene in the mantle cultured in medium with or without the AP-1 inhibitor SR11302. (**a-e**) Relative expression of *KRMP*, *N19*, *Pearlin*, *Prisilkin39*, and *Nacrein* mRNAs. The results were analyzed by t-test. Two asterisks means p < 0.01, and three asterisks means p < 0.0001.

**Figure 6:**
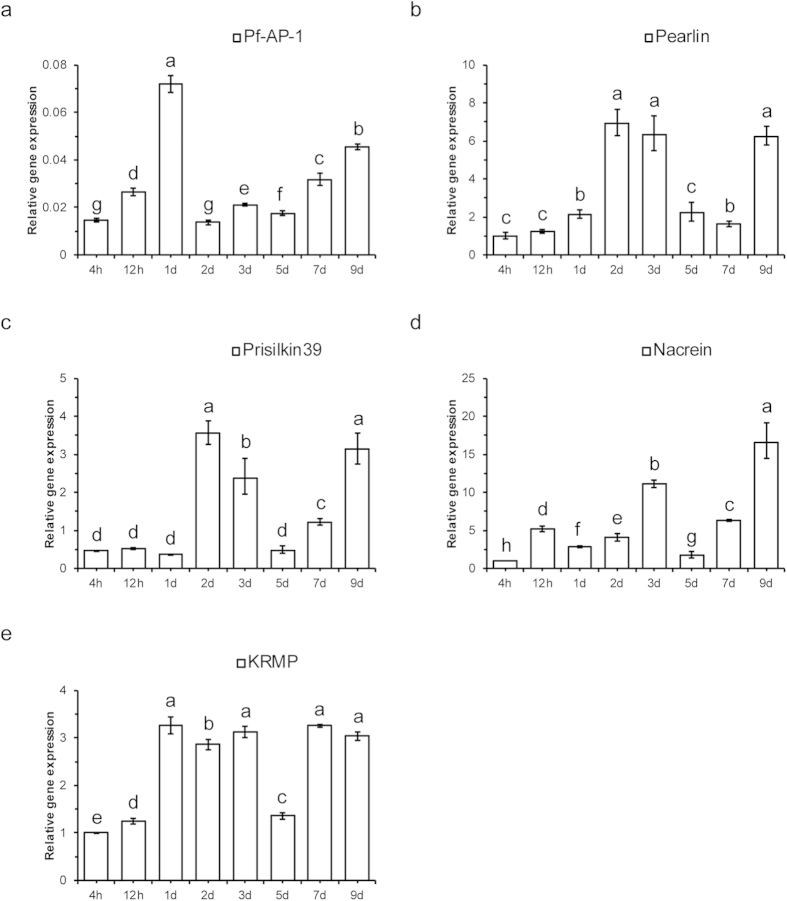
Relative mRNA expression of *Pinctada fucata* activator protein-1 (*Pf*-AP-1) and biomineral proteins during shell regeneration. Quantitative reverse transcription-polymerase chain reaction (qRT-PCR) analysis was performed to evaluate the expression of each gene at different time points. Abscissa shows time after notching. (**a–e**) Relative expression of *Pf-AP-1*, *Pearlin*, *Prisilkin39*, *Nacrein*, and *KRMP* mRNAs in the mantle near the shell breach. The results were analyzed by Duncan’s new multiple range method. Different superscript in the same figure means significantly different (p < 0.05).

We used AP-1 activity inhibitor SR11302 to stimulate the mantle tissue and determined the gene expression of matrix proteins. The results showed that AP-1 inhibitor could significantly depressed the expression of multiple matrix proteins such as KRMP, N19, Pearlin and Prisilkin39 (Fig. 5a–d). But the inhibitor had no effect on the expression of Nacrein (Fig. 6e). These results verified the *Pf*-AP-1 transactivation ability on matrix proteins.

### Gene expression patterns of *Pf-AP-1* and biomineral proteins during shell regeneration

The mantle was among the tissues with the highest *Pf*-AP-1 expression, revealing its potential function in biomineralization; thus, *Pf*-AP-1 gene expression levels were investigated during shell regeneration and pearl sac development. Gene expression levels of the biomineral proteins were also determined by qRT-PCR, and the Duncan’s new multiple range method was used to measure the significance of the different among each time points. In the shell regeneration experiment, we found that *Pf*-AP-1 expression increased to reach the maximum within 4 h to 1 day after artificial shell etching, decreased on day 2, and then increased inconsistently thereafter (Fig. 6a). Pearlin and Prisilkin39 expression had a similar pattern to that of *Pf*-AP-1, but the first expression peak appeared on day 3 (Fig. 6b,c). The first Nacrein expression peak appeared on day 5 after artificial shell etching. However, KRMP expression increased from 4 h to the end of day 1 and was maintained at a high level until day 5 (Fig. 6e).

### Gene expression patterns of *Pf-AP-1* and the biomineral proteins during pearl sac development

**Figure 7:**
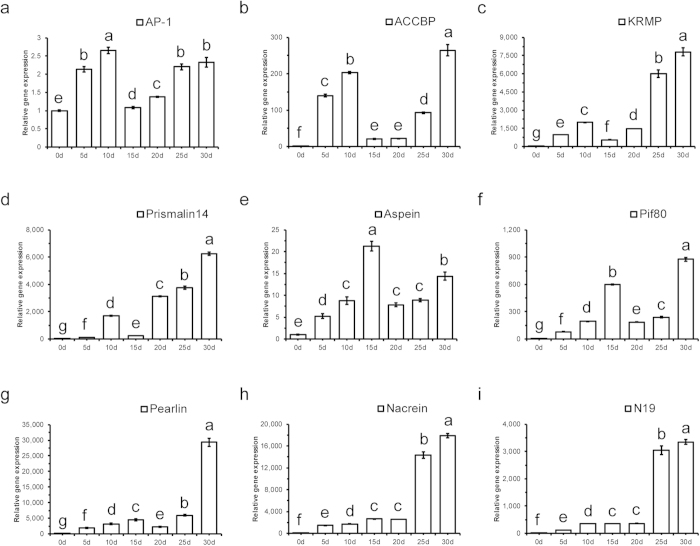
Relative mRNA expression of *Pinctada fucata* activator protein-1 (*Pf*-AP-1) and biomineral proteins during pearl sac development. Quantitative reverse transcription-polymerase chain reaction (qRT-PCR) analysis was performed to evaluate the expression of each gene at different development stages. Abscissa shows the time after implantation. (**a–i**) Relative expression of *Pf-AP-1*, *ACCBP*, *KRMP*, *Prismalin14*, *Aspein*, *Pif80*, *Pearlin*, *Nacrein*, and *N19* mRNAs in the pearl sac. The results were analyzed by Duncan’s new multiple range method. Different superscript in the same figure means significantly different (p < 0.05).

Gene expression patterns of *Pf*-AP-1 and the biomineral proteins during pearl sac development. To make the correlation more clear, we performed Duncan’s new multiple range method to measure the significance of the different among each time points, different letters mean significant difference (P < 0.05). We found that *Pf*-AP-1 expression increased significantly to its highest level during the first 10 days of pearl sac development, decreased significantly to baseline on day 15, and then increased to a higher level again from days 15 to 30 (Fig. 7a). Similar patterns were found among ACCBP, KRMP and Prismalin14, whose expression had two significant increases at day 10 and day 30 and a significant decrease at day 15, but their maximum appeared on day 30 (Fig. 7b–d). The expression patterns of Aspein, Pif80 and Pearlin were also similar to that of *Pf*-AP-1 but their first peaked on day 15. They increased significantly during the first 15 days and between days 20 to day 30 (Fig. 7e–g). The maximum Pif80 expression was observed on day 30 (Fig. 7f). To Nacrein and N19, their expression patterns were the same but had little similarity with *Pf*-AP-1 (Fig. 7h,i)

## Discussion

In mollusks, it is lack of studies on the transcriptional regulation in biomineralization process. PfMSX is the only transcription factor in *P. fucata* which had been studied and demonstrated having ability to activate transcription of certain matrix protein^28^. As more and more matrix proteins had been cloned and characterized, the research in transcriptional regulatory network of these matrix proteins become apparently requisite.

Jun is a central component of all AP-1 complexes and forms homodimers and heterodimers with all other AP-1 family members. Different AP-1 components have different functions and physiological effects^30^. However, only Jun/AP-1 was characterized from *P. fucata*; thus, most of the AP-1 members are yet to be studied. Based on the significance of AP-1 in the regulation of gene expression, more research on the AP-1 family is needed to identify the molluscan biomineralization mechanism.

As a typical bZIP transcription factor, *Pf*-AP-1 had a highly conserved basic region and a leucine-zipper domain. The residues that contacted the DNA and the hydrophobic interface heptad repeat residues in the C-terminal were unanimous among the AP-1 proteins from *P. fucata* and other species, and the potential similar transactivation ability of *Pf*-AP-1 has been confirmed using the amino acid sequence and protein structure^31^,^32^. Post-transcriptional modification of AP-1 is also essential for its activating function. One of the most extensively documented mechanisms is activation of Jun through the JNK cascade. JNKs are activated by the MAPK cascade and translocated to the nucleus where they phosphorylate Jun on its N-terminal transactivation domain.

Phosphorylation activates Jun, reduces ubiquitination, and consequently stabilizes the protein^10^,^33^. We found that sites phosphorylated by PAK2, MAPK8, PLK3, DYK2, and GSK-β on mammalian AP-1 also occur in *Pf*-AP-1 in similar positions, and that neighboring sequences were highly conserved. *Pf*-AP-1 also had a larger molecular weight than that of the calculated molecular weight. These results indicate similar post-transcriptional modifications and regulation of the activating ability of the molluscan bivalve AP-1 homolog. The existence of similar signaling pathways, such as PAK and MAPK, was also indicated.

We considered both query coverage and identity when aligning the protein sequences. Query coverage is the coverage of two sequences when their identities are similar. Our results show that *Pf*-AP-1 established higher query coverage and identities with the AP-1 proteins from higher animals, particularly those in human AP-1 compared with AP-1 proteins from other bivalves. *Pf*-AP-1 had the shortest distance to the main vertebrate branch in the molluscan branch of the phylogenetic analysis. All of these results indicate that the closest phylogenetic relationship and conserved function were between *Pf*-AP-1 and mammalian AP-1.

The bivalve mantle, which is the most important biomineral tissue, expressed the highest levels of *Pf*-AP-1. Similar results have been reported for *Crassostrea hongkongensis* and *Haliotis discus discus*, suggesting a potential relationship between *Pf*-AP-1 and biomineralization. The significant positive correlation we found between *Pf*-AP-1 and N19, Prisilkin39, Pearlin, and KRMP suggests a biomineral protein transcription-regulating function for *Pf*-AP-1. In *P. fucata*, the out fold participates in the formation of prismatic layer while the outer epithelial cells in the mantle participate in the formation of nacreous layer, and they secretion related matrix proteins respectively^34^,^35^. The results of *in-situ* hybridization showed that *Pf*-AP-1 had a strong expression in inner epithelial cells of outer fold and the outer epithelial cells in the mantle, suggesting that *Pf*-AP-1 had similar location with matrix proteins and might have important function in regulating matrix proteins.

However, there were other biomineral proteins which were not correlated with *Pf*-AP-1 (Figure S1), such as Pif80, Prismalin14, or Nacrein; thus, they confirmed the gene specificity of *Pf*-AP-1 transactivation. These results confirmed the significant positive correlation among these biomineralization proteins and demonstrate activation of the biomineral protein promoters by *Pf*-AP-1.

The luciferase reporter assay results showed transactication ability of *Pf*-AP-1 to matrix proteins *in vitro*. And the luciferase reporter assay results of Pearlin promoter 5′ deletion confirmed the *Pf*-AP-1 regulation ability to matrix protein and suggested potential functional site in region −191 to −81 bp in Pearlin promoter. TESS analysis showed that there were 5 putative AP-1 binding sites in this region. However, this analysis was made based on the AP-1 binding sites of human but not on the mollusks. Whether the promoter of mollusk had conserved transcription regulation binding motif with those in mammals had not been reported. So further studies were needed and were in process. The AP-1 inhibition experiment supported the transactivation ability of *Pf*-AP-1 to KRMP, N19, Pearlin and Prisilkin39 *in vivo*. And the Nacrein had no response to AP-1 inhibitor. This result supported that Nacrein had no correlation with *Pf*-AP-1 and that the transcativartion ability of *Pf*-AP-1 was gene specific.

The shell regeneration experiment is a routine method to study the physiological activities during shell repair and formation. We found increased *Pf*-AP-1 expression from 4 h to 1 day after shell notching in the shell regeneration experiment. This finding may have been due to stimulation from injury that caused an immune reaction. The shell resists attack from external parasites or pathogens. Previous studies have reported that the mantle probably participates in immunity. As the response to bacterial infection, the expression of immune-related factors in the mantle had an increase in 48h after bacterial challenge^36^,^37^,^38^. These results suggest that increased *Pf*-AP-1 expression in the mantle is due to the immune response and shell repair following activation of biomineral protein transcription. We found a linear increase in *Pf*-AP-1 expression on days 5–9 after notching. A similar pattern was observed for Prisilkin39 and Nacrein expression. This period is a rapid shell regenerating stage, in which *Pf*-AP-1 may specifically activate several biomineral proteins. Pearlin expression increased from day 7 rather than from day 5, which may have been due to a transcription regulation delay. The KRMP expression pattern was less similar to that of *Pf*-AP-1 than the other above-mentioned proteins.

In artificial pearl culture, the biomineral proteins regulating calcium carbonate crystals at the pearl surface are secreted by the pearl sac. Thus, development of the pearl sac is of great significance to artificial pearl formation. We also found that ACCBP, KRMP, and Prismalin14 shared a highly similar expression pattern to that of *Pf*-AP-1 during pearl sac development. Aspein, Pif80, Pearlin, and Nacrein had similar expression patterns but their first expression peaks were 5 days later. We speculate that this was similar to the Pearlin regulation delay during shell regeneration. All of the proteins measured in the shell regeneration experiment and during pearl sac development are important during shell and pearl formation^39^,^40^,^41^. Nacrein, Pif80, Pearlin, N19 and ACCBP can modify nacre lamellae morphology by inhibiting aragonite precipitation or growth of undesired aragonite crystal faces^39^,^40^,^42^,^43^,^44^,^45^,^46^. Prisilkin39 and KRMP participate in formation of the calcitic prismatic layer as well as the highly structured shell framework^47^,^48^. Aspein also directly regulate the formation of aragonite crystals and play important roles in calcite precipitation in the prismatic layer^49^. Thus, *Pf*-AP-1 may be essential for pearl sac development and shell formation.

However, we also found some expression changes that were not well explained by activating *Pf*-AP-1, such as KRMP during shell regeneration and Nacrein and N19 during pearl sac development. Thus, other more specific or unknown but important transcription factors may regulate expression of these proteins. Nevertheless, the transactivation ability of AP-1 had a strong time-space specification and was cell- and tissue-specific, so it may show that a gene regulated by *Pf*-AP-1 in one physiological process has no response in another process. Thus, *Pf*-AP-1 may regulate different biomineral proteins by forming different AP-1 dimeric proteins and interacting with diverse coactivators or cofactors during the different biomineralization processes.

In conclusion, *Pf*-AP-1 had a highly conserved structure with known AP-1 family members and was highly expressed in tissues that were important during biomineralization. *Pf*-AP-1 expression was positively correlated with that of several biomineral proteins, and the luciferase reporter assay verified the transactivation ability of *Pf*-AP-1 to those proteins. *Pf*-AP-1 revealed distinct expression patterns during shell regeneration and pearl sac development, which were similar to different degrees to the expression patterns of several biomineral proteins. Our results suggest that *Pf*-AP-1 is an important transcription factor that regulates several biomineral proteins and participates in several biomineralization processes in the bivalve *P. fucata*.

## Materials and Methods

### Animals

Live adult pearl oysters, *P. fucata* (approximately 2 years old), were purchased from Guofa Pearl Farm, Beihai, Guangxi Province, China. The oysters were maintained in aerated 20 °C artificial seawater (3% salinity) for 3 days and were used for experiments when the shells were 5.5–6.5 cm in length and wet weights were 45–55 g.

### Pearl sac selection

Artificial nuclei were implanted in oysters near the gonad. After implanting the nuclei, the pearl sacs were isolated carefully from gonad tissue every 5 days from days 0 to 30, and the samples were stored in liquid nitrogen. Ten oysters were used in each treatment group.

### Induction of shell regeneration

A V-shaped notch was cut in close proximity to the adductor muscle. The notch damaged the prismatic layer and the margin of the nacre but not the mantle, so the body of the oyster was not harmed. The oyster was returned to the aquarium immediately, and five samples were collected at different time intervals. Mantle tissue approximately 0.5 cm^2^ from the notch was cut with a scalpel, rapidly frozen in liquid nitrogen, and stored. The experiment had been repeated for three times.

### AP-1 inhibition experiment

The mantle explant culture of *P. fucata* was performed as described in Gong *et al*.^50^, with some modifications. The mantle was cut and sliced as small as possible using sharp ophthalmic scissors. The chopped mantle was cultured in 12 well cell cultrue cluster with 1mL medium per well. In experiment group, the tissue was simulted with 0.28 μM AP-1 inhibitor SR110302 (Tocris Bioscience, Minneapolis, MN, USA) and held for 24 h. Then the tissue and primary cells were harvest without medium and RNA was extracted to measure the gene expression level.

### Nucleic acid sample preparation

Total RNA of each tissue sample was extracted with Trizol (Invitrogen, Carlsbad, CA, USA), according to the manufacturer’s protocol. The cDNA temples were obtained by reverse transcription with the PrimeScript® RT Reagent Kit (Takara, Dalian, China) and used for molecular cloning and real-time PCR. The 3′ rapid amplification of cDNA ends (RACE) technique and 5′ RACE were performed using a BD SMART RACE cDNA Amplification Kit (Clontech, Palo Alto, CA, USA) to clone the complete mRNA.

### *In situ* hybridization

Gene-specific sense probe and antisense probe were used to exactly localize the expression of *Pf*-AP-1 in the mantle. The primers for the amplification of the inserted fragment into pGEM-T easy vector (Promega, Madison, USA) were showed in Table S1. Digoxigenin labeled RNA probes were synthesized from the linearized plasmid using DIG RNA Labeling Mix (Roche Diagnostics, Indianapolis, IN, USA) and T7 and SP6 RNA polymerase (Promega). The mantle was removed from the adult *P. fucata* and immediately fixed in 4% paraformaldehyde containing 0.1% DEPC (Sigma-Aldrich, St. Louis, USA) overnight. *In situ* hybridization was carried out on frozen sections of the mantle using the Enhanced Sensitive ISH Detection Kit II (AP) (Boster, Wuhan, China) as described previously^47^.

### Cloning the *Pf*-AP-1 CDS

The primers were designed based on the *Pf*-AP-1 fragments in the *P. fucata* mantle expression sequence tag library. The 5′- and 3′-ends were amplified using the SMARTer RACE cDNA Amplification Kit (Clontech), according to the manufacturer’s protocol. The PCR reactions were carried out using mantle cDNA as the template. A secondary nested PCR was carried out for higher specificity using the diluted primary PCR product as the template. The complete CDS was obtained by assembling overlapping fragments. The primers used for cloning are shown in Table S1. The ORF of the complete CDS was analyzed using ORF finder (http://www.ncbi.nlm.nih.gov/projects/gorf/). The deduced amino acid sequence structure was predicted by comparison with AP-1 homologs from other species. Phylogenetic relationships were analyzed using MEGA 5.22 software^51^ with the neighbor joining method^52^.

### Plasmid construction and antibodies

The pGL3basic and pcDNA3.1 plasmids were purchased from Promega. A FLAG tag sequence was inserted before the multiple cloning sites of the pcDNA3.1 plasmid, and the complete *Pf*-AP-1 CDS was subcloned into the plasmid to construct the FLAG-AP-1-pcDNA3.1 expression vector. The KRMP, Pearlin, and Prisilkin39 promoter was subcloned into pGL3basic. The primers used are shown in Table S1. The 5′ deleted promoter fragment used to construct Pearlin promoter deletion variant were amplified by a series of forward primers. The numbers in the name of primers mean the start site of the fragment in promoter. The primers are also shown in Table S1. The anti-FLAG antibody (M2) was purchased from Sigma-Aldrich (St. Louis, MO, USA).

### Cell culture, transfection, and immunoblotting

HEK293T cells were cultured in Dulbecco’s modified Eagle’s medium supplemented with 10% fetal bovine serum at 37 °C in a humidified 5% CO_2_ incubator. The HEK293T cells were seeded in 12-well plates 24 h before transient transfection. The transfection was mediated with VigoFect (Vigorous Biotechnology, Beijing, China), according to the manufacturer’s instructions.

Harvested cells were washed with phosphate-buffered saline for immunoblotting and then lysed in TNE buffer (50 mM Tris, pH 7.5; 0.5% Nonidet P-40, 150 mM NaCl, 1 mM EDTA, and protease inhibitors) at 4 °C. Total cell lysates were analyzed by sodium dodecyl sulfate-polyacrylamide gel electrophoresis and immunoblotting and detected with a chemiluminescent HRP substrate (Merck Millipore, Darmstadt, Germany), following the manufacturer’s instructions. The green fluorescent protein (GFP) was transfected into HEK293T as the blank control.

### Luciferase reporter assay

The luciferase reporter assay was performed using VARIOSKAN FLASH (Thermo Scientific, Waltham, MA, USA). HEK293T cells were transfected with the constructed plasmid and harvested 24 h later for the luciferase assay. The cells were co-transfected with the *Renilla* luciferase reporter plasmid (pRL-CMV, Promega) to normalize transfection efficiency.

### Semi-quantitative RT-PCR and qRT-PCR

Semi-quantitative RT-PCR was performed with gene-specific primers (Table S1). The LightCycler®480 II System (Roche) was used for qRT-PCR with the SYBR Premix Ex Taq II (Tli RNaseH Plus) (Takara). Cycle threshold (Ct) values were calculated for each reaction and normalized to an internal control (β-actin), and relative gene expression was calculated using the comparative Ct method^53^,^54^,^55^.

### Statistical analysis and transcription factor binding site prediction

T-test and the Duncan’s new multiple range method were used to measure the significance of different. In t-test results, one asterisk means p < 0.05, two asterisks means p < 0.01, and three asterisks means p < 0.0001. In the results of Duncan’s new multiple range method, different superscript means significantly different (p < 0.05).

Transcription factor binding sites were predicted by Transcription Element Search System (TESS), and the database of mammalian transcription factors was used in the analysis^56^.

## Additional Information

**How to cite this article**: Zheng, X. *et al*. The AP-1 transcription factor homolog *Pf*-AP-1 activates transcription of multiple biomineral proteins and potentially participates in *Pinctada fucata* biomineralization. *Sci. Rep*. **5**, 14408; doi: 10.1038/srep14408 (2015).

## Supplementary Material

Supplementary Information
