# Supplementary material for: The AP-1 transcription factor homolog Pf-AP-1 activates transcription of multiple biomineral proteins and potentially participates in Pinctada fucata biomineralization
Source: Sci Rep. 2015 Sep 25;5:14408. doi: 10.1038/srep14408 (PMC4585884; doi:10.1038/srep14408)

# The AP-1 transcription factor homolog *Pf*-AP-1 activates transcription of multiple biomineral proteins and potentially participates in *Pinctada fucata* biomineralization

Xiangnan Zheng^1+^ ∙Minzhang Cheng^1+^ ∙ Liang Xiang^1^ ∙ Jian Liang^1^ ∙ Liping Xie^1,2*^ ∙ Rongqing Zhang^1,2*^

**Figure S1** Relevance of *Pinctada fucata* activator protein-1 (*Pf*-AP-1), *Pif80*, *Prismalin14,* and *Nacrein* mRNA expression in normal oysters (n = 15), as analyzed by t-test.


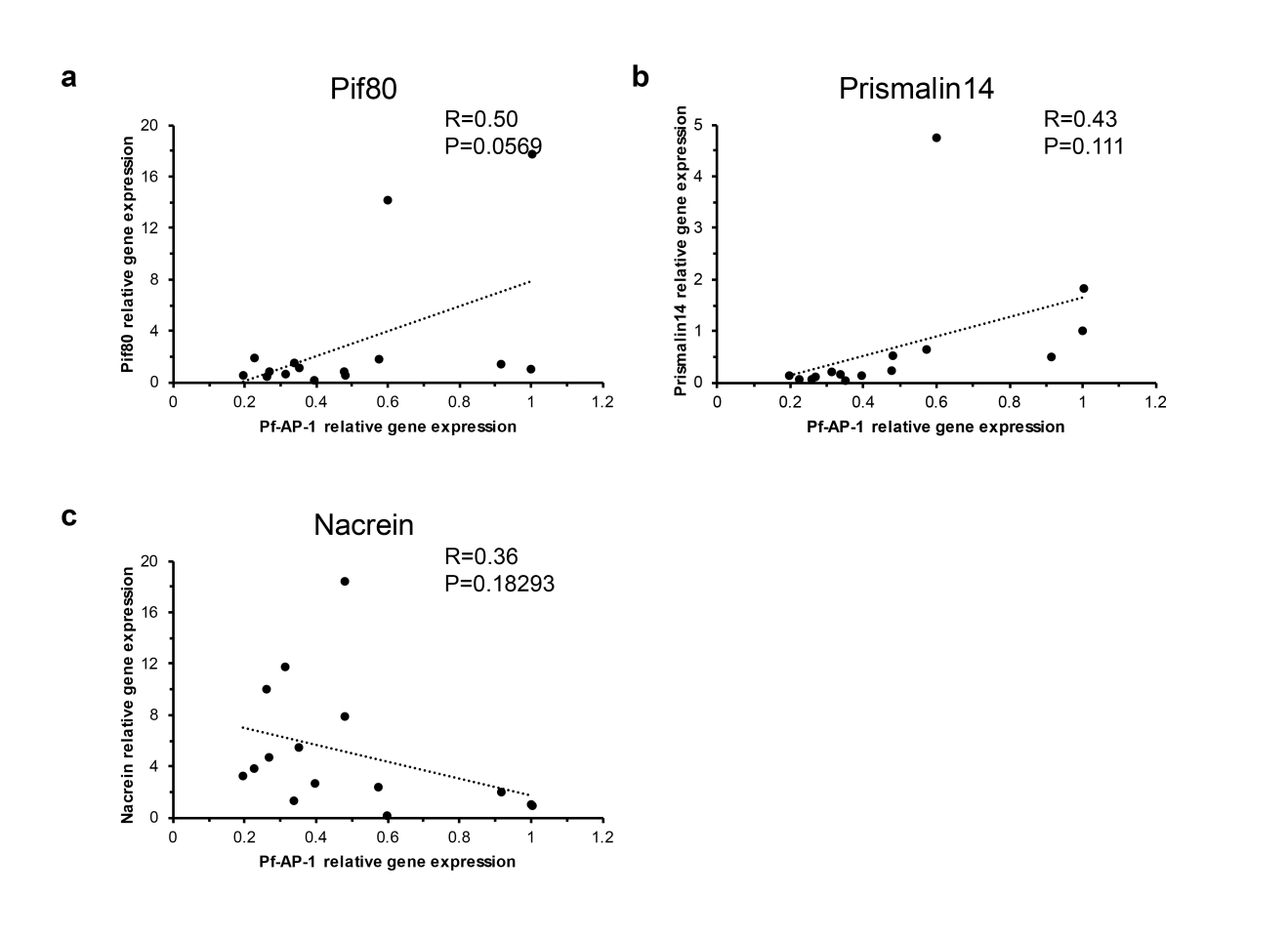


**Table S1** Sequences of the primers used for cloning, plasmid construction, and semi-quantitative and quantitative reverse transcription-polymerase chain reaction (qRT-PCR) analyses.


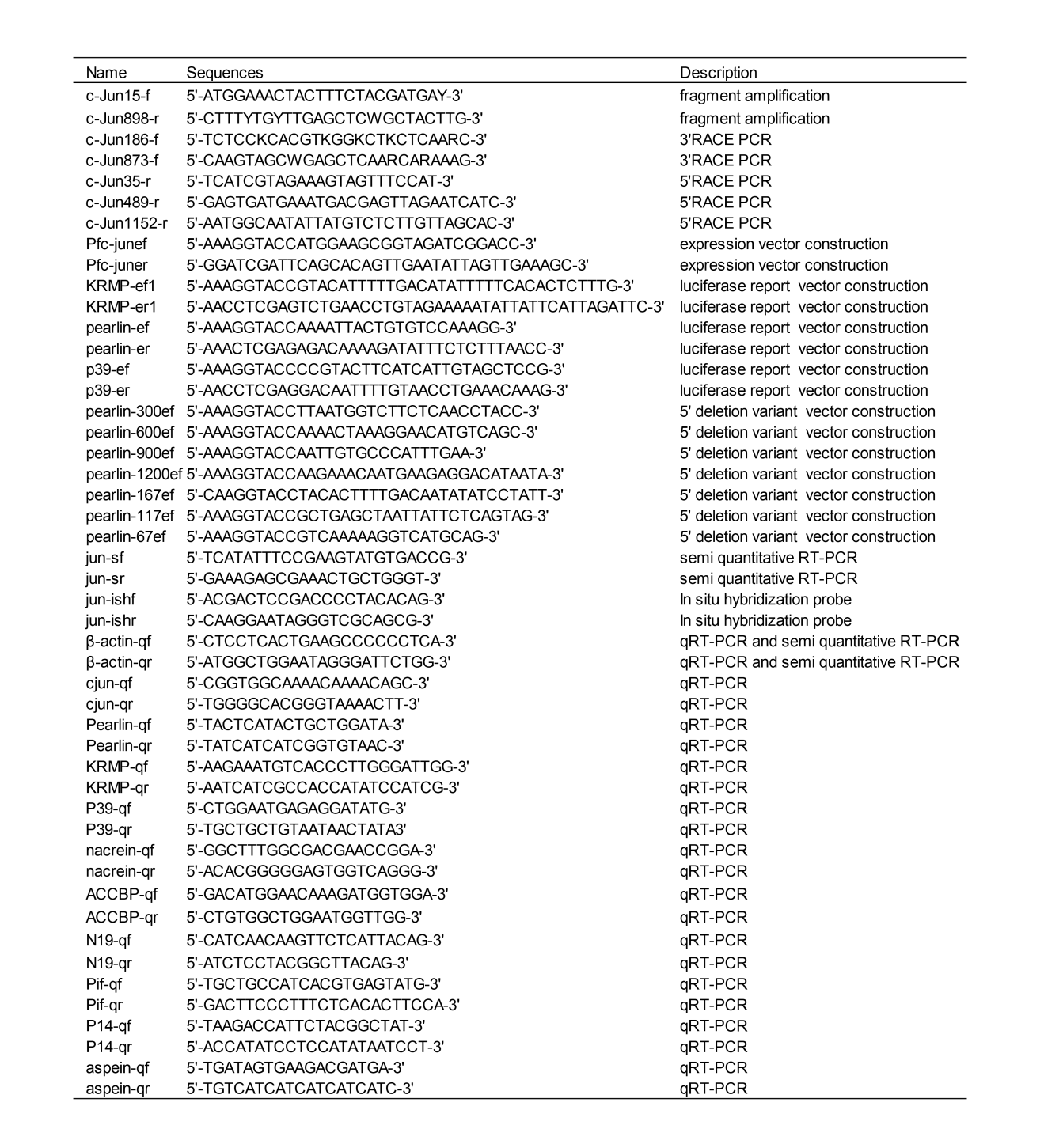

Supplement: Supplementary Information [file srep14408-s1.doc]
